# AAV-Mediated GALC Gene Therapy Rescues Alpha-Synucleinopathy in the Spinal Cord of a Leukodystrophic Lysosomal Storage Disease Mouse Model

**DOI:** 10.3389/fncel.2020.619712

**Published:** 2020-12-23

**Authors:** Michael S. Marshall, Yazan Issa, Gregory Heller, Duc Nguyen, Ernesto R. Bongarzone

**Affiliations:** Department of Anatomy and Cell Biology, College of Medicine, University of Illinois at Chicago, Chicago, IL, United States

**Keywords:** proteinopathies, alpha-synuclein, spinal cord, globoid cell leukodystrophy, Parkinson's disease

## Abstract

Krabbe's disease (KD) is primarily a demyelinating disorder, but recent studies have identified the presence of neuronal protein aggregates in the brain, at least partially composed by alpha-synuclein (α-syn). The role of this protein aggregation in the pathogenesis of KD is largely unknown, but it has added KD to a growing list of lysosomal storage diseases that can be also be considered as proteinopathies. While the presence of these protein aggregates within the KD brain is now appreciated, the remainder of the central nervous system (CNS) remains uncharacterized. This study is the first to report the presence of thioflavin-S reactive inclusions throughout the spinal cord of both murine and human spinal tissue. Stereological analysis revealed the temporal and spatial accumulation of these inclusions within the neurons of the ventral spinal cord vs. those located in the dorsal cord. This study also confirmed that these thio-S positive accumulations are present within neuronal populations and are made up at least in part by α-syn in both the twitcher mouse and cord autopsied material from affected human patients. Significantly, neonatal gene therapy for galactosylceramidase, a treatment that strongly improves the survival and health of KD mice, but not bone marrow transplantation prevents the formation of these inclusions in spinal neurons. These results expand the understanding of α-syn protein aggregation within the CNS of individuals afflicted with KD and underlines the tractability of this problem via early gene therapy, with potential impact to other synucleinopathies such as PD.

## Introduction

Krabbe's disease (KD) is a lysosomal sphingolipidosis caused by mutations in the galactosylceramidase gene (*GALC*) (Suzuki and Suzuki, 1971) leading to accumulation of the neurotoxic lipid galactosylsphingosine (or psychosine) (Svennerholm et al., 1980; Igisu and Suzuki, 1984). This results in central and peripheral nervous system demyelination, motor and neurosensory deficits, paralysis, and premature death, often before 2 years of age (Krabbe, 1916; Escolar et al., 2006; Barczykowski et al., 2012). Recently, neuronal protein aggregates have been identified within the brains of KD patients and within the twitcher mouse model (Smith et al., 2014). These aggregates contain, at least in part, alpha-synuclein (α-syn) (Smith et al., 2014). Protein aggregates of α-syn are a pathological hallmark of certain adult-onset neurodegenerative diseases such as Parkinson's disease (PD) (Spillantini et al., 1997) and have been identified in other lysosomal storage disorders, most notably Gaucher's disease (GD) (Shachar et al., 2011; Xu et al., 2011). The significance of this pathological commonality between PD and Gaucher is supported by the findings that mutations in the *GBA1* gene that lead to GD are one of the strongest risk factors for PD (Sidransky et al., 2009; Bultron et al., 2010; Siebert et al., 2014). Multiple other lysosomal genes have been identified as risk loci for PD including *GALC* (Chang et al., 2017; Li et al., 2018), supporting a possible similar connection between KD and PD. Additionally, dysfunction in psychosine metabolism caused by *GALC* mutations has been suggested as a risk factor for PD (Marshall and Bongarzone, 2016; Marshall et al., 2018a).

The significance of these protein aggregates within KD is not well-understood; however, there is a temporal and spatial correlation between α-syn and psychosine accumulation in the brain, which follows analogous patterns observed in some PD patients (Smith et al., 2014). Importantly, we showed that a neonatal gene therapy using adeno-associated viral vectors to express GALC (AAV9-GALC) is capable of preventing the formation of these protein aggregates in the KD mouse brain (Abdelkarim et al., 2018). Mechanistically, psychosine binds to α-syn and can promote an aggregation-prone configuration of the protein (Abdelkarim et al., 2018). The relevance of this interaction was supported after genetic knock-out of the α-syn *SNCA* allele in twitcher mice, which showed mild motor and cognitive improvements (Abdelkarim et al., 2018). To date, the aggregates have only been identified and studied within the brain of individuals affected with KD. However, PD patients have been shown to not only have α-syn inclusions located within the brain but also throughout the spinal cord (Braak et al., 2007; Nardone et al., 2007; Del Tredici and Braak, 2012; VanderHorst et al., 2015). Whether similar spreading to the spinal cord of KD individuals occurs remains unaddressed.

Based on our studies showing α-syn accumulations in the twitcher brain (Smith et al., 2014) and their positive response to AAV-based gene therapy (Abdelkarim et al., 2018), here we investigated the hypothesis that the entire CNS and therefore the spinal cords of twitcher and human Krabbe patients are also impacted by neuronal protein aggregates, and are responsive to gene therapy correction of GALC deficiency.

## Methods

### Mouse and Human Tissue

Animals used in this study were done in accordance with the approved protocols (Protocol#18-072) from the Animal Care and Use Committee at The University of Illinois at Chicago. Twitcher (GALC -/-) mutant mice and WT (GALC+/+) mice, maintained on a C57BL/6J background, were identified by PCR as previously described (Sakai et al., 1996). Non-identified human tissue was obtained from the University of Maryland Brain bank (Cases 575 and 1163 and controls 1547 and 4247).

### Gene Therapy and Bone Marrow Transplantation

Description of animals used in this study has been previously detailed in (Marshall et al., 2018b). Males and females were indistinguishably used in this study. Mice (*n* = 6 per group) were randomly distributed as treatment was initiated before sex could be identified. Neonatal TWI mice (P0-P1) were treated with AAV9-GALC *via* three delivery routes (intracranial, I.C., intrathecal, I.T., and intravenous I.V.) for a total dosage of 4.2 × 10^11^ vg per mouse (indicated as TWI+AAV) as we described. After 24 h of recovery, a subset of mice (indicated as TWI+AAV+BMT) were subjected to bone marrow transplantation BMT) and given an additional I.V. injection of bone marrow stem cells harvested from 6 to 8 weeks old syngeneic WT mice (30 million cells each treatment). An additional group (BMT only) received only the I.V. injection of bone marrow stem cells, also harvested from 6 to 8 weeks old syngeneic WT mice (30 million cells each treatment). Last, a group of sham (sterile PBS) I.T., I.V., and I.C. injections on TWI and WT were also perform on P1-P2 days of age. After treatments, mice were returned to their home cage with mother. Any treated mice that died within 2 days of injections were excluded from this study.

### Galactosylceramidase Activity and Psychosine Quantification

Our methods have been described in detail in (Marshall et al., 2018b). Briefly, fresh frozen tissue was homogenized in H_2_O using a Vibra-cell ultrasonic liquid processor model#VCX 130 (Sonics and Materials Inc., Newton, CT). Tissue lysates (20 μg) were incubated with fluorescent GALC substrate (6HMU-beta-D-galactoside; Moscerdam Substrates) for 17 h at 37°C before the reaction was stopped. Enzymatic activity was assessed *via* fluorescence using a Beckmann Coulter DTX 880 multimode detector (Beckman Coulter, Brea, CA) using excitation/emission wavelengths of 385 and 450 nm, respectively. Psychosine was extracted from tissue homogenates (200 μg) *via* a methanol-acetic acid solution (0.5% Acetic Acid in methanol). Using D-lactosyl-ß1-1'-D-*erythro*-sphingosine cat#860542P (Avanti Polar Lipids, Alabaster, AL) as an internal standard, psychosine content was determined using tandem mass spectrometry.

### Immunohistochemistry and Thioflavin-S Staining

Mice were anesthetized and perfused with saline followed by 4% paraformaldehyde before tissue was removed and processed for cryosectioning. Cryosections (30 μm) were blocked free-floating with blocking buffer (0.3 M glycine, 1% BSA, 5% normal donkey serum, 5% normal goat serum, 0.30% Triton X-100, TBS) for 1 h at room temperature, followed by 24–72 h incubation at 4°C with primary antibodies in blocking solution. After washing with TBS, tissue was incubated with secondary antibodies at room temperature for 1 h in blocking buffer and washed again in TBS. Finally, sections were incubated in 0.005% thioflavin-S (cat# T1892, Sigma, St. Louis, MO) for 8 min, followed by two washes in 50% ethanol for 1 min and a TBS wash. Tissue was mounted with Prolong Gold antifade reagent (cat# P36931 Life technologies, Eugene, OR) and visualized using confocal microscopy (Leica TCS SPE, Wetzlar, Germany). Primary antibodies used included: α-syn (mouse) (cat# 610787 BD Biosciences, San Jose, CA, 1:300 dilution) and NeuN (rat) (cat# MAB377 Millipore, Darmstadt, Germany 1:250 dilution). Secondary antibodies used included: AlexaFluor 488 anti-Mouse (cat#A-11029, Thermo Fisher Scientific, Waltham, MA, 1:500 dilution) and Dylight 549 anti-rat (cat# 112-506-068 (Jackson ImmunoResearch, 1:500 dilution). Counterstaining for cell nuclei was performed with DAPI (cat#D1306, Thermo Fisher Scientific, Waltham, MA, 1: 3,000 dilution in TBS). Immunofluorescent complexes were visualized using a Leica TCS SPE confocal laser with an upright DM5500Q Microscope (Leica Biosystems Inc., Buffalo Grove, IL).

### Stereological Quantification

For stereological quantification, the spinal cord (*n* = 3 per group per time point) was perfused and fixed with 4% paraformaldehyde. The cervical, thoracic (not included in this study), and lumbar regions were separated based on anatomical landmarks of the adjacent vertebrae. The cervical and lumbar regions were then completely sectioned on a microtome into 50-μm sections and kept in order in labeled containers with cryoprotectant solution. Sequential 50-μm sections of the entire cervical or lumbar spinal cord for each genotype were stained for thioflavin-S which included incubation in 0.005% thioflavin-S (cat# T1892, Sigma, St. Louis, MO) for 8 min, followed by two washes in 50% ethanol for 1 min and a TBS wash. The tissue was mounted with Prolong Gold antifade reagent (cat# P36931 Life technologies, Eugene, OR). Once mounted, spinal cord sections were divided into a ventral and dorsal half with the division line passing through the central canal. Quantification of cell soma with thioflavin-S positive material was performed with a design-based stereology system (Stereo-Investigator version 8, MBF Bioscience, Williston, VT, USA), using tracing under a 5X objective and counting under a 63X objective (Zeiss AX10 microscope, Carl Zeiss Ltd., Hertfordshire, England). The sampling parameters were set up per the software guide to achieve the coefficient of error ranged between 0.09 and 0.12 using the Gundersen test, normally with a counting frame size 100 × 100 μm, optical dissector height 35 μm, and an average of 10 sampling sites per section.

### Statistical Analysis

Statistics and graphs were prepared with Prism 8 software (GraphPad Software Inc., La Jolla, Ca). Data with more than two means being compared were analyzed using a one-way ANOVA with Gaussian distribution, with two-sided *p*-values <0.05 considered significant. Comparisons between two means were analyzed with *t*-tests, with two-sided *p* < 0.05 considered significant. Graphs represent the mean of independent measurements (with sample sizes ranging *n* = 2–4) shown with errors bars representing standard error of the mean.

### Data Availability

All original data presented in this study is available upon request.

## Results

### Thioflavin-S Positive Inclusions Progressively Accumulate in the Gray Matter of the Spinal Cord of Twitcher Mice With Regional Variability

**Figure 1:**
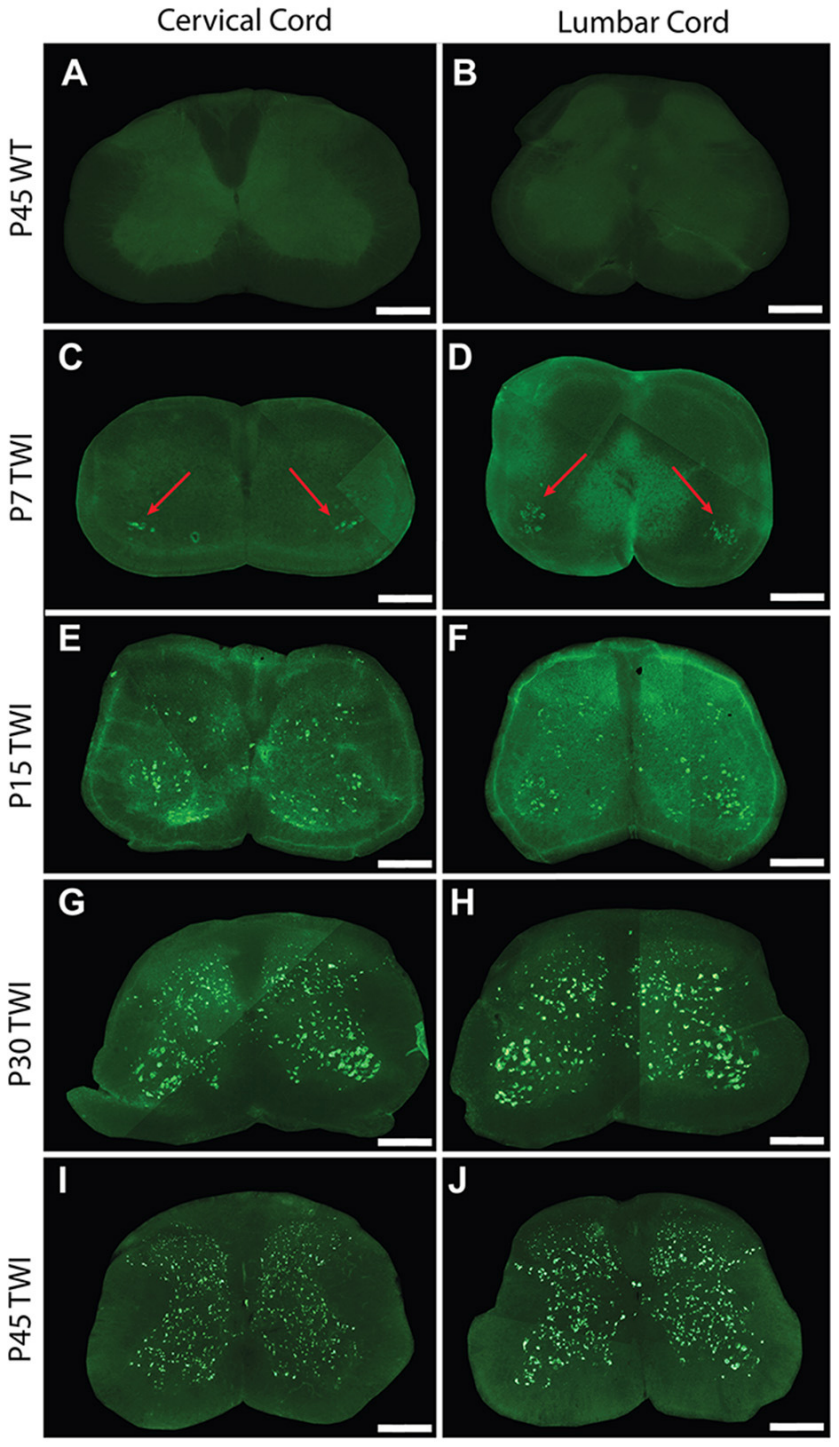
Thioflavin-S accumulation in spinal cord across life span. Spinal sections from the cervical cord of post-natal day 7 (P7) **(C)**, P15 **(E)**, P30 **(G)**, and P45 **(I)** twitcher mice were stained with thioflavin-S (thio-S) to reveal presence of protein inclusions. There is a progressive accumulation across lifespan with the earliest detection at P7 primarily present only in the ventral horn (**C, red arrows**). A similar pattern of accumulation was noted within the lumbar horns at the same time-points P7 (**D, red arrows**), P15 **(F)**, P30 **(H)**, and P45 **(J)**. Cervical **(A)** and lumbar **(B)** spinal sections from a P45 wild type (WT) mouse reveal the background staining and confirm a lack of thio-S reactive material. Images depicted are representative of the thio-S inclusion burden within the cervical and lumbar regions of the spine. They do not represent a precise spinal level. Scale bars represent 0.5 mm.

To investigate the spatial and temporal accumulation of protein aggregates that may be present in the spinal cord, sections from the cervical and lumbar levels of the cord from twitcher mice were stained for the presence of thioflavin-S (thio-S) positive inclusions. At post-natal day 7 (P7), these thio-S inclusions were present almost exclusively in the anterior gray column (lamina VIII and IX) of the ventral horns of both the cervical (Figure 1C, red arrows) and lumbar cord (Figure 1D, red arrows). Occasional sparse inclusions in the dorsal horn were sometimes noted as well without an identifiable predilection for specific gray matter lamina. P7 was the earliest analyzed timepoint that identified these inclusions with no thio-S positive inclusions found at post-natal days 1 and 4 (data not included). At P15, thio-S inclusions were consistently present in both the dorsal and ventral horns in the spinal cord at both the cervical and lumbar levels (Figures 1E,F). The distribution of thio-S inclusions became more diffuse throughout both horns and at all cord levels at P15, with an increase in density. The thio-S inclusion density continued to increase as the mouse aged to P30 (Figures 1G,H) and furthermore at P45 (Figures 1I,J), at which point the aggregates were widely disseminated throughout the gray matter of the spinal cord. These aggregates were not identified within the white matter of the spinal cord. For comparison, thio-S staining in WT spinal tissue is at background levels as shown (Figures 1A,B).

### Stereological Quantification Confirms Regional and Temporal Differences in the Accumulation of Thioflavin-S Inclusions Across Twitcher Lifespan

**Figure 2:**
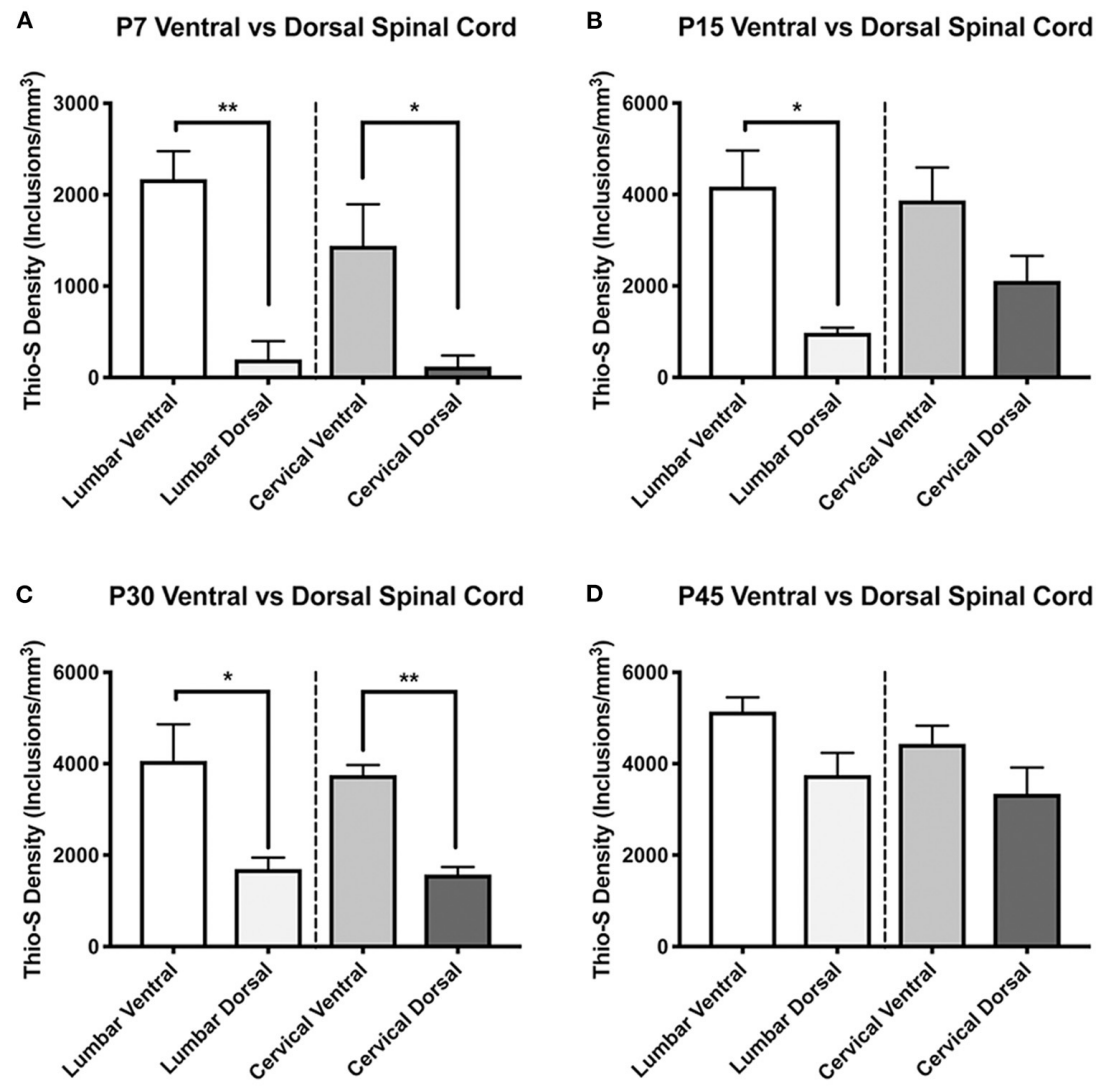
Thioflavin-S quantification and comparison between ventral and dorsal spinal cords across life-span. Stereological analysis was performed to quantify thioflavin-S (thio-S) accumulation and compare thio-S density (inclusions/mm^3^) between ventral and dorsal spinal cord **(A–D)**. Significantly more thio-S inclusions were observed in the ventral cord as compared to the dorsal at all timepoints **(A–C)** expect post-natal day 45 (P45) **(D)**. Significance between means analyzed by ANOVA and Tukey's *post-hoc* analysis with (*) indicating *p* < 0.05, (**) *p* < 0.01. *n* = 3–4 animals. Results are presented as mean ± error of the mean.

Subsequently, we quantified the density of the thio-S positive inclusions using stereological analysis. As there appeared to be a difference in the timing of inclusion accumulation between the ventral and dorsal horns, we analyzed the spinal cord with the dorsal and ventral cord as separate regions. Regional distribution of inclusions between the ventral and dorsal spinal cord was delineated with a division through the central canal. This quantification confirmed the histological observations that inclusions are more prevalent in the ventral cord at P7 compared to the dorsal cord (Figure 2A), showing a significant (*p* < 0.05) increase in both the lumbar and cervical cords. This significant difference was observed in the lumbar cord at P15 and P30 (Figures 2B,C, respectively) and in the cervical cord at P30 (Figure 2C) but not P15 (Figure 2B). Neither level of spinal cord found any significant differences between the ventral and dorsal cord at P45 (Figure 2D), demonstrating an equilibration of inclusion density between ventral and dorsal cord as the animals aged.

**Figure 3:**
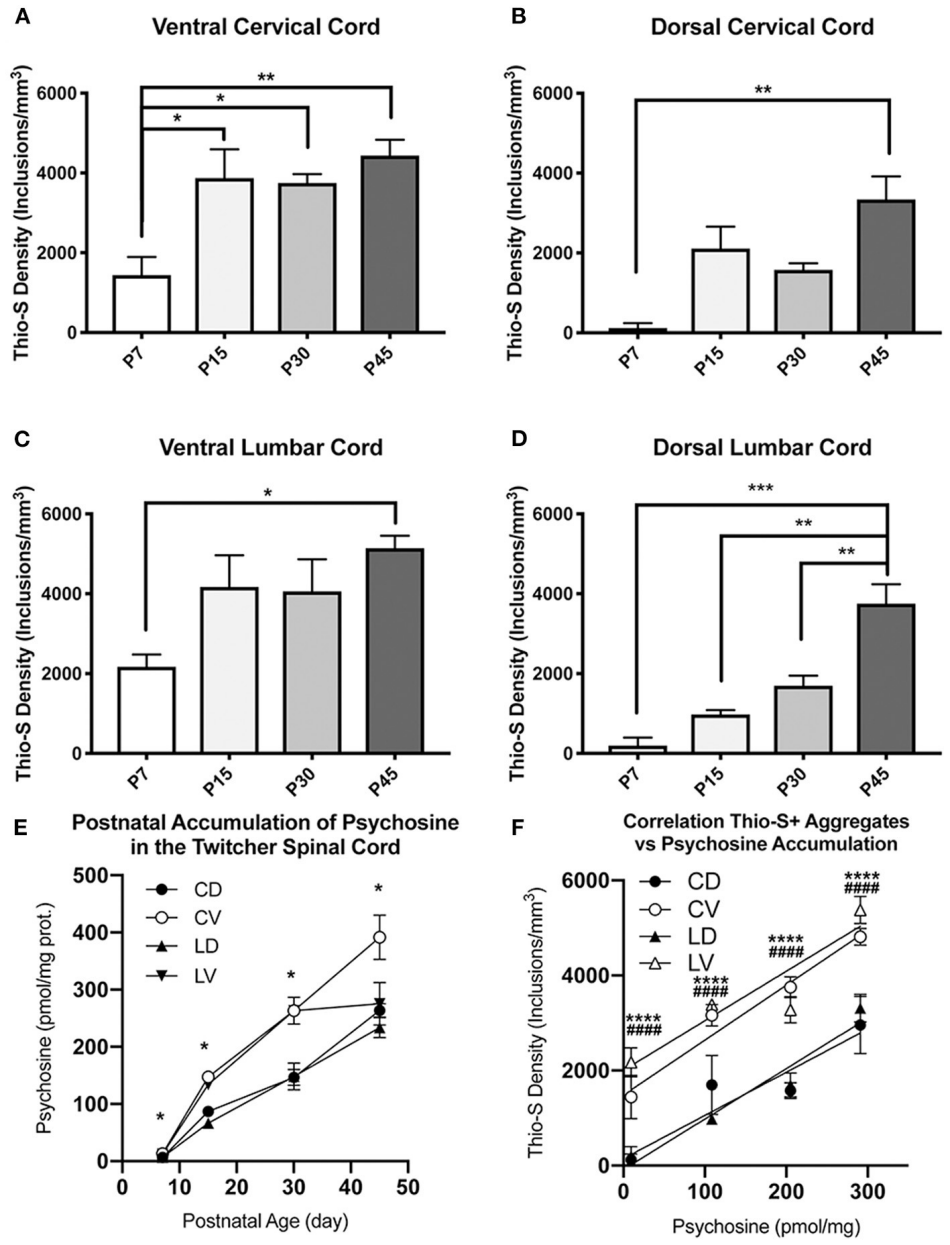
Quantification and comparison of thioflavin-S accumulations across life-span. Stereological analysis of thioflavin-S accumulation density (inclusions/mm^3^) was performed. The ventral cervical and lumbar cords revealed large early accumulations at P7 and P15 with relatively slight additional accumulation over the final two time points **(A,C)**. The dorsal spinal cord **(B,D)** showed a more protracted accumulation across life span. Psychopsine levels were measured in the cervical ventral (CV), cervical dorsal (CD), lumbar ventral (LV), and lumbar dorsal (LD) cord regions. This was compared across post-natal development **(E)** and in correlation to thio-s accumulation **(F)**. **(A–D)** Significance between means analyzed by ANOVA and Tukey's *post-hoc* analysis with (*) indicating *p* < 0.05, (**) *p* < 0.01, and (***) *p* < 0.001, *n* = 3–4 animals. **(E,F)** Significance between means analyzed by ANOVA and Tukey's *post-hoc* analysis with (*) indicating *p* < 0.05 and (****) *p* < 0.0001 in comparison between CV and CD; (####) *p* < 0.0001 in comparison between LV and LD, *n* = 2–3 animals. Results are presented as mean ± error of the mean.

To visualize whether this equilibration represents a decline in the rate of ventral inclusion accumulation or conversely an increase in accumulation in the dorsal cord, the data was compared between age groups. This confirmed the significant accumulation of thio-S inclusions that occurs over the animals' lifespans (P7–P45) (Figures 3A–D). The increases between time points in the two regions suggest that due to the ventral cord being disproportionately affected at an early age, there may be a plateauing effect, limiting the rate that accumulation can occur in the ventral cord. This can be seen by the rapid increase in thio-S density from P7 to P15 ages within the ventral cord, which then has very mild and non-significant increases in thio-S densities from P15 to P30 and P45. To support this explanation, P45 animals were double stained with thioflavin-S and the neuronal marker NeuN. The density of thio-S inclusions could then be normalized to the number of neurons present in the ventral and dorsal spinal cord. This analysis found significantly higher concentrations of thio-S inclusions per number of neurons in the ventral cord at P45 due to the higher number of neurons counted within the dorsal region compared to the ventral region (Supplementary Figure 1). Thus, although at P45 the difference in the number of affected neurons between the ventral and dorsal cords is non-significant, this appears to be due to a limiting factor of fewer neurons present in the ventral cord that could continue to develop thio-S inclusions.

To further elucidate this difference in thio-S inclusion accumulation between the ventral and dorsal spinal cord, psychosine was measured within each cord region (Figure 3E). Within the cervical and lumbar cord, the ventral halves had higher levels of psychosine at each timepoint measured. In the cervical cord, this increase reached statistical significance. Interestingly, when the level of psychosine was correlated to the density of thio-S inclusions, there was a significantly higher density of inclusions in the ventral spinal cord for both the cervical and lumbar regions (Figure 3F), even at an early post-natal timepoint (P7) when psychosine accumulation is relatively low. These results further support that the ventral spinal cord is particularly vulnerable to developing thio-S inclusions compared to the dorsal cord.

### Thioflavin-S Inclusions Present Only in Neurons in the Spinal Cord and Colocalizes With Alpha Synuclein

**Figure 4:**
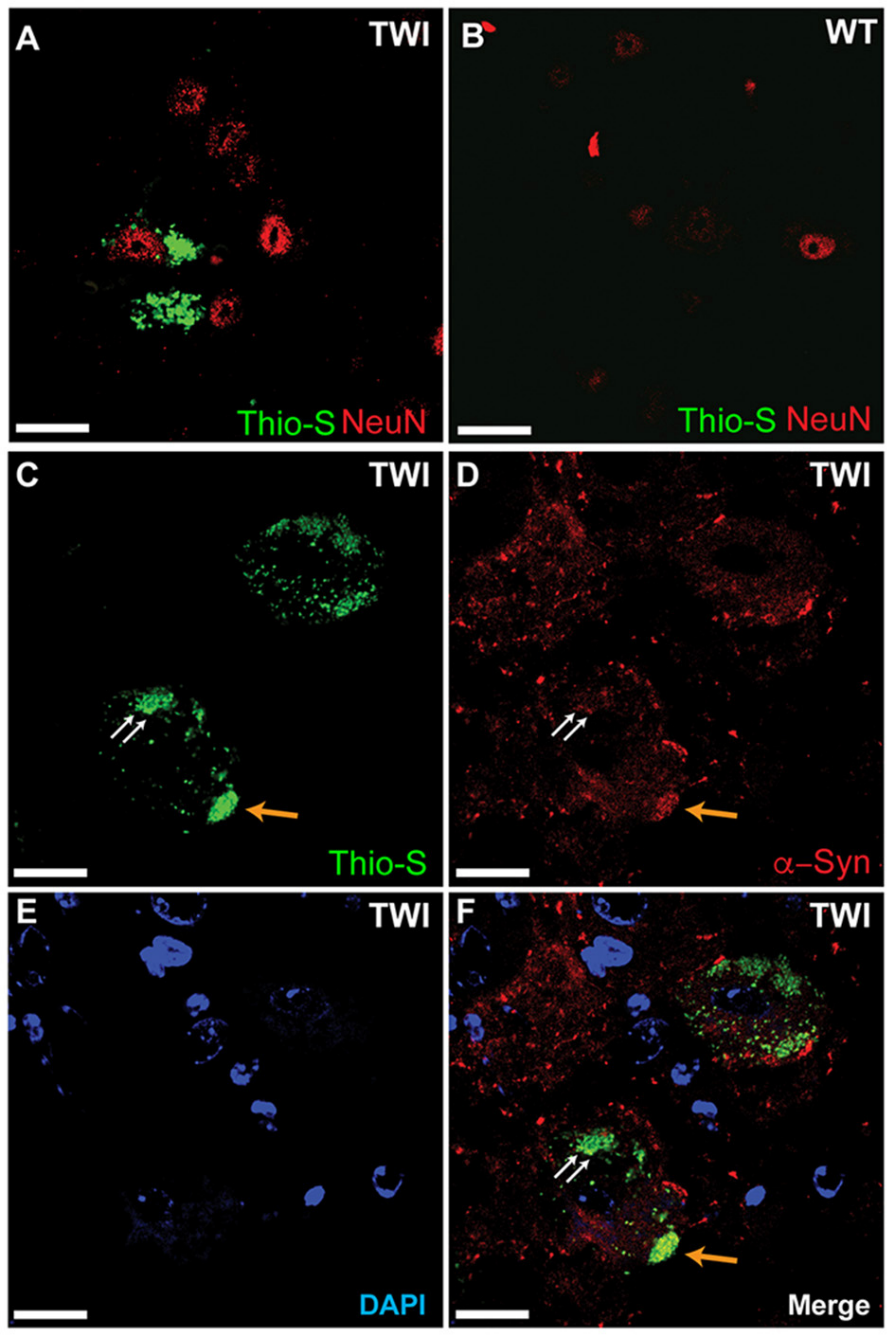
Thioflavin-S accumulations present in neuronal cell populations of spinal cord. Spinal tissue from TWI **(A)** and WT **(B)** animals was stained with thioflavin (thio-S) and the neuronal marker NeuN, which found thio-S inclusions within neurons of TWI but not within neurons of WT animals. Dual-staining in TWI with thio-S **(C)** and alpha-synuclein (α-syn) **(D)** revealed colocalization **(F)** of a-syn with the thio-S inclusions. Cell nuclei are highlighted by DAPI **(E)**. Scale bars represents **(A–F)**: 20 μm.

We examined spinal tissue dual-stained with thioflavin-S and NeuN in both TWI (Figure 4A) and WT (Figure 4B) mice. Consistent with previous analysis in brain tissue (Smith et al., 2014), we observed thio-S inclusions within somas of cells positive for the neuronal marker NeuN in TWI tissue (Figure 4A). Markers for oligodendrocytes, astrocytes, and microglia did not reveal thio-S inclusions within those cell populations, which is also consistent with analysis in the brain (Smith et al., 2014) (data not shown). We next investigated if these protein aggregations contain α-syn as previously identified in the brain (Smith et al., 2014). Dual-staining with thio-S (Figure 4C) and α-syn (Figure 4D) did identify areas of colocalization (Figure 4F, arrows), consistent with previous reports (Smith et al., 2014). Cell nuclei were also identified with DAPI staining (Figure 4E).

### Thioflavin-S/Alpha-Synuclein Inclusions Observed in Human Spinal Cord Tissue

**Figure 5:**
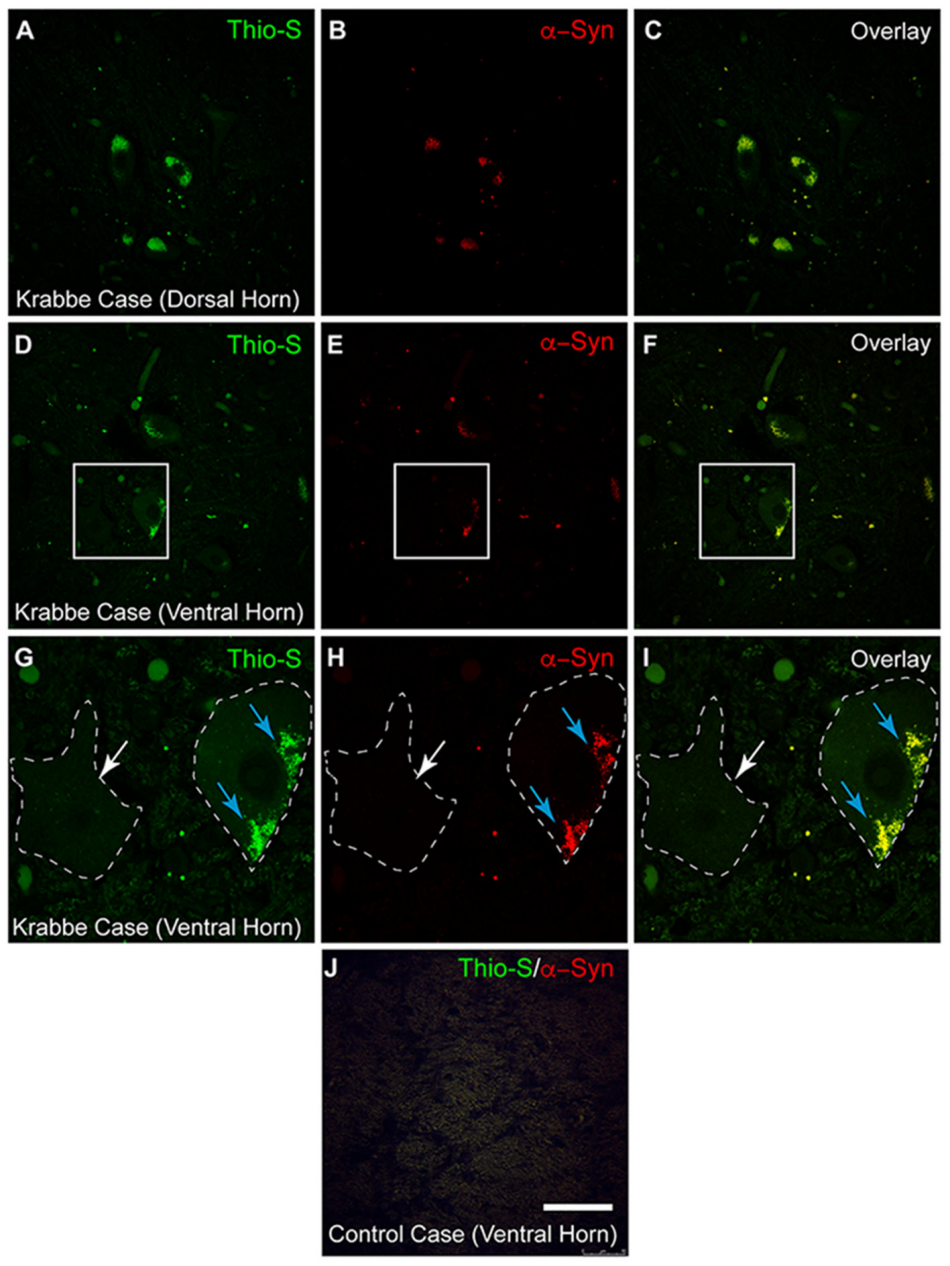
Alpha-synuclein staining in human Krabbe disease patients. Human spinal tissue from a patient with Krabbe **(A–F)** was stained with thioflavin-S **(A,D)** and for α-synuclein (α-syn) **(B,E)**. Overlay of the two channels demonstrated colocalization of thioflavin-S reactive material with α-syn in cells of both the dorsal horn **(C)** and ventral horn **(F)**. **(G–I)** [corresponding to the boxes in **(D–F)**, respectively] show a cell with thioflavin-S and α-syn positive material as highlighted by the blue arrows compared to an unaffected cell highlighted by the white arrow. Cell comparison between panels is highlighted by white dashed line. Affected patients were both 2 years of age and in terminal stage of disease. Last, spinal tissue stained concurrently from an unaffected control case is shown in **(J)**. Scale bar represents **(A–F)**: 40 μm; **(G–J)**: 20 μm.

To confirm that these observations are not unique to the twitcher mouse model, spinal tissue from two human Krabbe cases (each aged 2 years and in terminal stage of the disease) was also stained with thioflavin-S and α-syn (Figures 5A–F). Thio-S positive material was identified in both the dorsal horns (Figure 5A) and ventral horns (Figure 5D) of the Krabbe cases. Co-staining for α-syn (Figures 5B,E) and overlay of the two channels (Figures 5C,F) confirmed the colocalization of the thio-S positive inclusions with α-syn. A cell affected by cytoplasmic inclusions of thio-S/α-syn material (highlighted by blue arrows) is shown adjacent to an unaffected cell (highlighted by a white arrow) in Figures 5G–I (each of which correspond to the white boxes in Figures 5D–F, respectively). Last, tissue from an age-matched control infant was stained for thioflavin-S and α-syn, which did not contain any cellular inclusions (Figure 5J).

### Early Treatment of Twitcher Mice With AAV9-GALC Gene Therapy but Not BMT Prevents the Formation of Spinal Thioflavin-S Inclusions

We have previously shown that thioflavin-S positive inclusions in the TWI brain can be attenuated with an adeno-associated virus (AAV)-9-based gene therapy protocol to replace the deficient GALC enzyme (Marshall et al., 2018b). Here we investigated whether the thioflavin-S inclusions in the spine were also responsive to AAV-GALC gene therapy. TWI mice were treated at an early post-natal age (P0-P1) with one of three treatment regimens: AAV-GALC gene therapy delivered via combined intracranial, intravenous, and intrathecal injections, intravenous BMT from syngeneic WT mouse, or a combination of the two therapies. Tissue was collected at age P40, which is near the typical maximum lifespan of an untreated TWI mouse and at an aged timepoint. This aged timepoint was collected once the animal died or was euthanized per a predetermined set of humane endpoint criteria.

**Figure 6:**
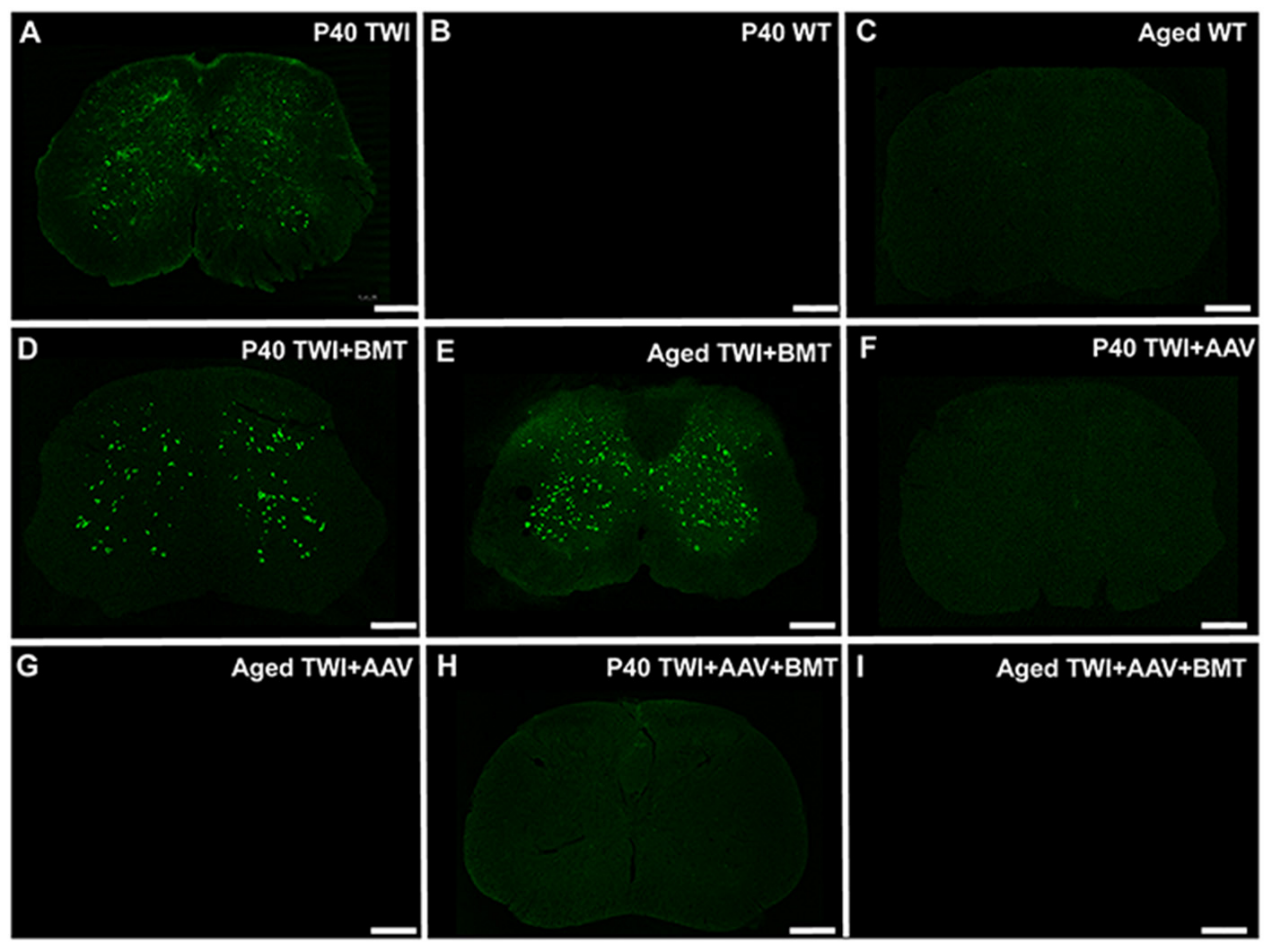
Thioflavin-S positive inclusions are prevented with AAV-GALC gene therapy but not bone marrow transplantation. The extent of thio-S positive inclusions in the TWI **(A)** spinal cord, was not significantly improved with bone marrow transplantation (BMT) at either a P40 **(D)** or an aged (85 days of age) **(E)** timepoint. Treatment with AAV gene therapy either alone **(F,G)** or in combination with BMT **(H,I)** prevented the development of thio-S inclusions at both an early (P40) and late (aged, 350–550 days of age) timepoint. The staining present in the AAV and AAV+BMT treated TWI was comparable to WT mice at the same timepoints **(B,C)**. Observations present in *n* = 3 animals for all groups except aged TWI-AAV which *n* = 2. Scale bars represent 0.5 mm.

Compared to untreated TWI (Figure 6A), Thio-S staining in BMT treated TWI at either P40 (Figure 6D) or an aged timepoint (Figure 6E) showed no significant difference in the level of thio-S staining. When TWI were treated with AAV, thio-S positive inclusions were not identified at either P40 (Figure 6F) or an aged timepoint (Figure 6G), comparable to a WT animal at similar timepoints (Figures 6B,C). TWI treated with a combination of AAV and BMT revealed the same results with no inclusions identified at either timepoint (Figures 6H,I). Spinal tissue from P40 sham treated TWI along with tissue from aged WT and treated TWI were examined for GALC activity and psychosine content. TWI, which have no measurable GALC activity at baseline, had a significant increase after AAV-GALC therapy with a mean level of GALC activity higher than that found in the aged WT spine (Supplementary Figure 2A). This correlated to a significant reduction in psychosine content by both treatment regimens with AAV (Supplementary Figure 2B). AAV-GALC gene therapy under this protocol has previous been shown to restore GALC activity and normalize psychosine levels in the brain (Marshall et al., 2018b). Additionally, previous reports showed this correction of GALC activity and psychosine levels was most effective with a combined intrathecal, intracranial, and intravenous delivery and is why it was chosen for this study rather than a single intrathecal delivery of AAV-GALC (Marshall et al., 2018b). TWI treated with BMT-only showed no significant increase in GALC activity. The BMT-only treatment group did result in a significant decrease in psychosine levels compared to sham treated P40 TWI. However, the psychosine level remained high and was significantly higher than aged WT animals. These results support that correction of the underlying GALC deficiency and therefore normalization of psychosine levels can be effective in preventing the formation of thio-S+/α-syn spinal neuronal inclusions.

## Discussion

When Knud Krabbe first described “a diffuse sclerosis of the brain” in 1916, later to be known as Krabbe disease (KD), he also noted that “the medulla and spinal cord show extensive loss of axis cylinders and their medullary sheaths especially affecting the pyramidal tracts (Krabbe, 1916; Compston, 2013).” Even with this early acknowledgment of KD's spinal involvement, there is a notable dearth of studies performed to understand whether there are unique characteristics in the pathogenesis of KD within the spinal cord (Castelvetri et al., 2011, 2013; Cantuti-Castelvetri et al., 2012). The importance of which is highlighted by therapeutic studies that have found a targeted delivery to the spinal cord leads to an optimal response (Karumuthil-Melethil et al., 2016; Marshall et al., 2018b). Therefore, with the recent discovery that the brains of twitcher mice and human KD patients are affected by neuronal inclusions composed at least in part by α-syn (Smith et al., 2014), the chief aim of this study was to characterize whether a similar phenomenon is present within the spinal cord of individuals afflicted of this lysosomal storage disease.

This study identified thio-S positive protein inclusions that are composed of, at least in part, α-syn within spinal neurons of both the twitcher mouse and human KD patients. The small, punctate pattern of staining found in this study is consistent with prior reports that revealed this pattern to be due to the subcellular localization of the α-syn inclusions within the lysosome (Smith et al., 2014; Abdelkarim et al., 2018). While the patterns of staining observed here between the TWI and humans tissue are similar, comparisons between the pattern of staining in the murine tissue and that from human KD patients is difficult due to diverse epitope properties of murine and human alpha-synuclein (Dhillon et al., 2017). The inclusions were first identifiable within the ventral horn of the spinal cord, with stereological analysis finding a significantly increased density of inclusions within the ventral half of the spinal cord at early timepoints (P7–P30) in comparison to dorsal spinal cord that later equilibrated in the last measured timepoint of the animals' lives (P45). Normalization of these inclusions to the number of neurons suggested that the ventral spinal cord neurons maintain a significantly increased propensity for accumulation of protein inclusions throughout the animals' lifespans. The distribution of protein inclusions has been attributed to differences in the regional accumulation of psychosine as the level of psychosine has been shown to correlate with the density of protein inclusions within the brain (Smith et al., 2014). Further, psychosine has been demonstrated to induce aggregation of α-syn and induces an aggregation prone conformation through direct binding of psychosine to α-syn (Smith et al., 2014; Abdelkarim et al., 2018). In this study, we did find that the ventral cord had significantly higher levels of psychosine compared to the dorsal cord at each timepoint measured. However, we found that the ventral cord had significantly higher thio-S/α-syn inclusions throughout post-natal development, even at early timepoints (P7) when the amount of psychosine accumulation is relatively low. This further supports the regional vulnerability of the ventral spinal neurons to accumulate these protein inclusions at an accelerated and more robust rate.

The reason for this regional difference in psychosine and protein inclusion accumulation is unknown. It may be due to differential production of α-syn between the ventral and dorsal spinal cord neurons or there may be pro-aggregating factors in the local environment of the ventral neurons that are absent or minimal in the dorsal cord. For instance, one possibility may be due to differences in the rate of myelination production and turnover in spinal cord. In murine development, oligodendrocyte maturation has been shown to be more robust in the ventral cord (Foran and Peterson, 1992). However, myelin breakdown and globoid cells have been described to be more concentrated within the dorsal columns of the twitcher mouse (Takahashi and Suzuki, 1984) and a higher concentration of PAS-positive macrophages was observed in the dorsal funiculus of the canine model of KD (Fletcher et al., 1977). Yet, in these studies myelin breakdown and globoid cells were not detected until post-natal day 25 and became pronounced throughout the entire spinal white matter only after day 40 (Takahashi and Suzuki, 1984). This dichotomy between the most affected areas of white and gray matter suggests there may be intrinsic cellular factors responsible for the neuronal dysfunction rather than being solely attributable to changes in the cells' environment. Mechanisms specific to neuronal dysfunction in KD have been described and suggested to play a role in the motor, sensory and cognitive deficits that develop in Krabbe patients (Castelvetri et al., 2011, 2013; Smith et al., 2011; Cantuti-Castelvetri et al., 2012). These same mechanisms may contribute to or be in part due to these protein inclusions. For instance, α-syn has been observed to be trafficked by axonal transport (Freundt et al., 2012; Brahic et al., 2016), which could explain the aggregation of α-syn in the neuronal cell body secondary to axonopathy and axonal transport deficits noted in KD (Castelvetri et al., 2011, 2013).

Due to the limited availability of post-mortem brain tissue from KD patients, only two patients were able to be analyzed, but both were found to have the presence of thio-S+/α-syn aggregates. Additional studies will hopefully confirm these results are generalizable to the entire KD patient population, Still, the presence of protein inclusions composed of α-syn has raised the possibility of KD as a synucleinopathy (Smith et al., 2014; Marshall and Bongarzone, 2016). Another lysosomal storage disorder, Gaucher disease, has been found to not only be affected by α-syn aggregates (Manning-Bog et al., 2009; Mazzulli et al., 2011; Xu et al., 2011; Yap et al., 2011; Siebert et al., 2014), but mutations in Gaucher's causative gene (*GBA1*) have been identified as one of the strongest risk factors for developing PD (Sidransky et al., 2009; Bultron et al., 2010; Barrett et al., 2013; Siebert et al., 2014; Aflaki et al., 2017). While the association is not as strong, mutations in *GALC* have also been associated with PD (Chang et al., 2017; Li et al., 2018; Marshall et al., 2018a). Within synucleinopathies such as PD and multiple system atrophy (MSA), spinal α-syn accumulation has been identified, particularly within the intermediolateral columns of the thoracic and sacral cord, and portions of gray matter layers 1,7, and 9 (Braak et al., 2007; Nardone et al., 2007; Sasaki et al., 2008; Del Tredici and Braak, 2012; VanderHorst et al., 2015). With the emerging evidence connecting lysosomal storage diseases such as Gaucher and KD to PD, the findings in this study are potentially relevant for understanding spinal accumulations within PD and other synucleinopathies.

In PD, patients often experience dysautonomia and pain disorders, in addition to the classic parkinsonian motor deficits (Chaudhuri et al., 2006; Jankovic, 2008). Given the distribution of α-syn in the spinal cord, it's believed that these spinal lesions play a role in these non-motor symptoms, including pain, impaired micturition, constipation, and orthostatic hypotension (Braak et al., 2007; Nardone et al., 2007; Del Tredici and Braak, 2012; VanderHorst et al., 2015). These spinal accumulations may also contribute to motor dysfunction as α-syn aggregates have also been identified within the pyramidal tracts of patients with PD and MSA (Yoshida, 2007). Additionally, increased α-syn accumulation in the ventral horn neurons of MSA patients is associated with increased incidence of myoclonus clinically (Hwang et al., 2019). Therefore, these newly identified lesions within the spines of the twitcher mouse and human KD patients may also play a role in both the motor and non-motor deficits such as dysautonomia, myoclonus, and irritability experienced by KD patients (Beltran-Quintero et al., 2019). Interestingly, the earlier and more dense accumulation of protein inclusions within the ventral horn neurons is consistent with the temporal pattern of symptoms observed in the twitcher, which includes significantly earlier development of motor deficits before sensory impairment is measurable (Olmstead, 1987).

The identification and characterization of these thio-S inclusions in the spine of mice and human patients affected by KD, not only provides a more complete understanding of the pathological changes in KD but will also hopefully lead to better therapeutic strategies. AAV based vectors are one of the most frequently used delivery techniques for investigating gene therapy approaches in KD (Marshall et al., 2018b) and are a commonly employed strategy for many other diseases. Currently, they are being used in over 200 ongoing clinical trials (Goswami et al., 2019). While these vectors are non-pathologic and generally safe, they are limited by the immunogenicity of their capsid proteins and the high prevalence of anti-AAV antibodies in humans (Goswami et al., 2019). Additionally, human trials require large doses of vector which is expensive and difficult to produce in large quantities (Goswami et al., 2019). To address these limitations, preclinical studies have aimed at developing targeted protocols that can efficiently deliver the vector while still achieving satisfactory disease correction. An increasingly recognized effective strategy is the use of intrathecal administration of gene therapy in KD (Reddy et al., 2011; Qin et al., 2012; Karumuthil-Melethil et al., 2016; Marshall et al., 2018b; Bradbury et al., 2020). Multiple studies have achieved significant improvements in lifespan and motor function with protocols that deliver a single intrathecal gene therapy dose (Karumuthil-Melethil et al., 2016; Bradbury et al., 2020). This study is an important step toward characterizing the full extent of KD pathology in the spinal cord and supports the importance of intrathecal delivery for treating KD with gene therapy.

Our results demonstrate that using AAV-GALC gene therapy to correct GALC activity in TWI and normalize psychosine levels at a neonatal age prevents the formation of thio-S+/α-syn inclusions. Previous studies have found these inclusions to be closely associated with the regional psychosine content of the brain (Smith et al., 2014) and that psychosine binds to α-syn and can promote an aggregation-prone configuration of the protein (Abdelkarim et al., 2018). Together, these findings support psychosine accumulation in the spinal cord (Zhu et al., 2016) as a causal event for the formation of thio-S+/α-syn inclusions in the spinal cord of KD, a process which we show in this study, can be reversed with early AAV-GALC gene therapy. We also show that BMT in the mouse model did not prevent the formation of thio-S+/α-syn inclusions in the TWI spinal cord. As we have shown in the brain, solitary BMT therapy does not significantly increase GALC activity and fails to fully normalize psychosine levels (Marshall et al., 2018b). The lack of significant GALC and psychosine correction in the spine by BMT further supports this hypothesis. Whether hematopoietic stem cell transplantation (HSCT), currently utilized for treating patients with KD, would have similar results is unclear. The level of stable donor chimerism achieved under this therapy protocol without immunosuppressive preconditioning has been previously reported as 10.45% (Marshall et al., 2018b). In humans, the level of donor chimerism achieved is much higher and often complete (Allewelt et al., 2018), thus HSCT in humans may result in more effective prevention of α-syn inclusions.

The physiological impact of these inclusions in KD is not completely understood but knock-out of the α-syn allele, *SNCA*, in TWI has shown a modest but significant increase in lifespan along with improvements to cognitive and motor function (Abdelkarim et al., 2018). Further, the *GALC* gene has been identified as a risk loci for PD (Chang et al., 2017; Li et al., 2018), and dysfunction in psychosine metabolism by GALC has been suggested as a risk factor for PD (Marshall and Bongarzone, 2016; Marshall et al., 2018a). Given the evidence that GALC deficiency may play a contributing factor for the development of synucleinopathies in a subset of patients (Marshall and Bongarzone, 2016; Chang et al., 2017; Li et al., 2018; Marshall et al., 2018a), this therapy may have utility in these forms of late-onset synucleinopathies, beyond its use in KD. In this study, therapeutic correction of GALC was performed prior to any evidence of thioflavin-S inclusion formation. Therefore, it remains to be answered whether these inclusions can be cleared once they have become established. Preventing and potentially reversing the formation of α-syn aggregates is of great clinical interest, and these results provide an intriguing new therapeutic target for the treatment of some forms of synucleinopathies.

The discovery that α-syn neuronal inclusions are present within the spinal cord and are not exclusive to the brain, expands the understanding of KD as a possible demyelinating synucleinopathy. It provides a new mechanism for understanding neuronal vulnerability in KD and how these aggregates could potentially play a role in the sensory and motor deficits that KD patients experience. The early and rapid progression of the inclusions in the twitcher mouse provides a novel and potentially powerful model to study α-syn aggregation in the spinal cord. Additionally, the use of AAV-GALC gene therapy to prevent the development of these neuronal inclusions underscores the promise of this therapy to improve symptoms in not only KD patients but potentially a subset of patients affected by other synucleinopathies.

## Data Availability Statement

The original contributions presented in the study are included in the article/Supplementary Material, further inquiries can be directed to the corresponding author/s.

## Ethics Statement

The animal study was reviewed and approved by Animals used in this study were done in accordance with the approved protocols (Protocol#18-072) from the Animal Care and Use Committee at The University of Illinois at Chicago.

## Author Contributions

MM and EB designed the study and wrote the manuscript. MM, YI, and EB performed immunofluorescence and immunohistochemical staining, subsequent confocal imaging, and analyzed the data. YI performed stereological analysis. GH performed thioflavin-S analyses. DN produced animals for the study. All authors read and approved the manuscript.

## Conflict of Interest

EB is a consultant for Lysosomal Therapeutics Inc., E-Scape Bio, Gain Therapeutics, Affinia, and Neurogene. Neither entity provided support in the form of salaries for any listed author nor played additional roles in the study design, data collection, analysis, decision to publish, or preparation of the manuscript. The remaining authors declare that the research was conducted in the absence of any commercial or financial relationships that could be construed as a potential conflict of interest.

## Supplementary Material

Supplementary Figure 1 Thioflavin-S positive accumulations normalized to neuronal population. NeuN staining was used to identify neuronal cells. Number of thioflavin-S (thio-S) positive cells was normalized to the number of neuronal cells in both the lumbar and cervical spinal cord of P45 TWI. Significance between means analyzed by *t*-test with (^*^) indicating *p* < 0.05 and (^**^) *p* < 0.01. *n* = 2 animals. Results are presented as mean ± error of the mean.

Supplementary Figure 2 GALC activity and psychosine levels in spinal cord normalize after AAV-GALC gene therapy. Spinal tissues from post-natal day 40 (P40) twitcher (TWI) mice and aged WT and AAV gene therapy-treated TWI mice were analyzed for GALC activity **(A)**. Aged WT had a significantly higher level of GALC activity compared to P40 TWI. TWI treated with either AAV gene therapy alone or in combination with bone marrow transplantation (BMT) had a significantly higher level of GALC activity. TWI treated with BMT only did not show a significant increase in GALC activity. Tissue from the same animals was also analyzed for psychosine content **(B)**. P40 TWI were found to have significantly higher psychosine levels compared to aged WT and the aged AAV-treated TWI. TWI treated with BMT only had a significant decrease in psychosine levels compared to the P40 TWI but were still significant elevated compared to aged WT. Significance between means analyzed by ANOVA and Tukey's *post-hoc* analysis with (^****^) indicating *p* < 0.0001. *n* = 3 animals for all groups except aged TWI-AAV which *n* = 2. Results are presented as mean ± error of the mean.
