# Supplementary figures and images for: AAV-Mediated GALC Gene Therapy Rescues Alpha-Synucleinopathy in the Spinal Cord of a Leukodystrophic Lysosomal Storage Disease Mouse Model
Source: Front Cell Neurosci. 2020 Dec 23;14:619712. doi: 10.3389/fncel.2020.619712 (PMC7785790; doi:10.3389/fncel.2020.619712)

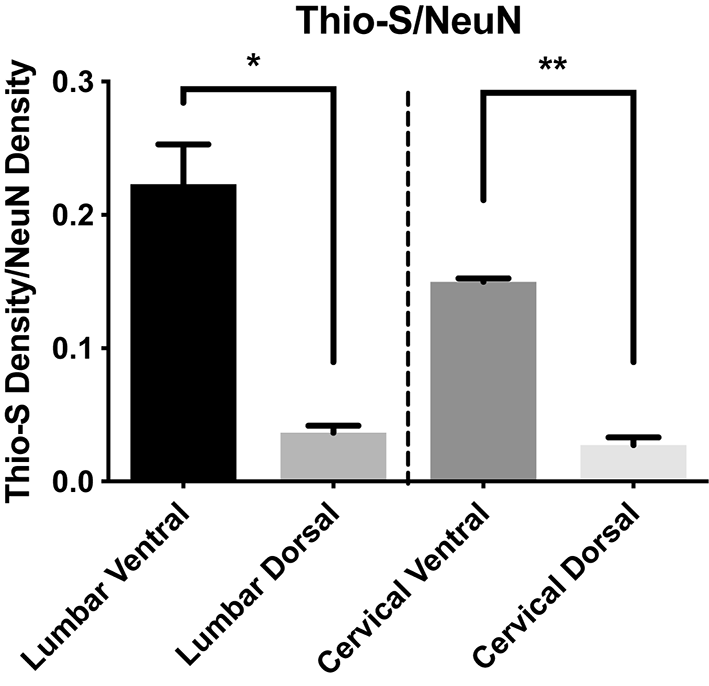

Supplement: Supplementary Figure 1 — Thioflavin-S positive accumulations normalized to neuronal population. NeuN staining was used to identify neuronal cells. Number of thioflavin-S (thio-S) positive cells was normalized to the number of neuronal cells in both the lumbar and cervical spinal cord of P45 TWI. Significance between means analyzed by t-test with (*) indicating p < 0.05 and (**) p < 0.01. n = 2 animals. Results are presented as mean ± error of the mean. [file Image_1.TIF]

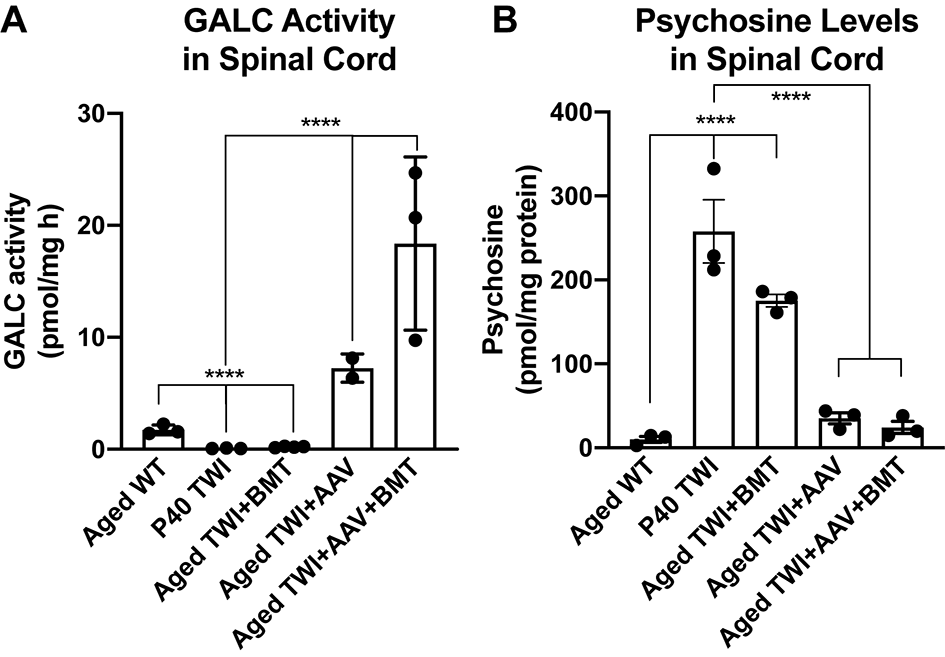

Supplement: Supplementary Figure 2 — GALC activity and psychosine levels in spinal cord normalize after AAV-GALC gene therapy. Spinal tissues from post-natal day 40 (P40) twitcher (TWI) mice and aged WT and AAV gene therapy-treated TWI mice were analyzed for GALC activity (A). Aged WT had a significantly higher level of GALC activity compared to P40 TWI. TWI treated with either AAV gene therapy alone or in combination with bone marrow transplantation (BMT) had a significantly higher level of GALC activity. TWI treated with BMT only did not show a significant increase in GALC activity. Tissue from the same animals was also analyzed for psychosine content (B). P40 TWI were found to have significantly higher psychosine levels compared to aged WT and the aged AAV-treated TWI. TWI treated with BMT only had a significant decrease in psychosine levels compared to the P40 TWI but were still significant elevated compared to aged WT. Significance between means analyzed by ANOVA and Tukey's post-hoc analysis with (****) indicating p < 0.0001. n = 3 animals for all groups except aged TWI-AAV which n = 2. Results are presented as mean ± error of the mean. [file Image_2.TIF]
